# Field Biology of the Beetle *Aegopsis bolboceridus* in Brazil, with a List of Host Plants

**DOI:** 10.1673/031.013.4801

**Published:** 2013-06-04

**Authors:** Charles M. Oliveira, Marina R. Frizzas

**Affiliations:** 1 Embrapa Cerrados, Rod. BR 020 km 18 (Brasília/Fortaleza), C. Postal 08223, Planaltina, DF, 73310-970, Brazil; 2 Universidade de Brasília, Departamento de Zoologia, Instituto de Ciências Biológicas, Brasilia/DF, 70910–900, Brazil

**Keywords:** Agaocephalini, behavior, bioecology, Cerrado, corn pest, Dynastinae, larval instars, life-cycle, vegetable pest, white grub

## Abstract

The white grub, *Aegopsis bolboceridus* (Thomson) (Coleoptera: Melolonthidae), is an important vegetable and corn pest in central Brazil. The objective of this study was to examine the biology of *A. bolboceridus* in the field and to update the list of its host plants. The study was conducted in an area with vegetable crops and corn located in the Federal District of Brazil. Samplings were taken to observe the biological stages of *A. bolboceridus*, preferred oviposition sites, and the adult swarming period. *A. bolboceridus* exhibited a univoltine cycle that lasted approximately 12 months from egg to active adults. Its eggs were found from October to November. The larval stage lasted approximately eight months, occurring between October and May. Pre-pupae were observed between April and June, and pupae were found between May and July. Inactive adults were observed in July and August, and the swarming period was between September and October. The females preferred to oviposit in sites with taller plants. Four new plant species were identified as hosts for this pest, and two new locations were recorded for its occurrence. This study is the first to describe the biology of a representative of the tribe Agaocephalini in Brazil.

## Resumo

A espécie *Aegopsis bolboceridus* (Thomson) (Coleoptera: Melolonthidae) é uma importante praga de hortaliças e milho na região central do Brasil. O objetivo desse trabalho foi estudar a biologia de *A. bolboceridus* em campo no Cerrado do Brasil Central e atualizar a lista de suas plantas hospedeiras. O estudo foi conduzido em uma área de produção de hortaliças e milho no Distrito Federal. Foram realizadas amostragens das fases biológicas de *A. bolboceridus* em campo, estudos sobre a preferência para oviposição e período de revoada dos adultos. *A. bolboceridus* apresentou ciclo univoltino que durou cerca de 12 meses de ovo a adulto. Os ovos foram encontrados em campo de outubro a novembre O estágio larval durou cerca de 8 meses, ocorrendo entre outubro e maio. As pré-pupas foram observadas entre abril e junho e as pupas entre maio e julho. Adultos inativos ocorreram em julho e agosto e em revoada em setembro e outubro. Fêmeas dessa espécie preferiram ovipositar em areas com plantas mais altas. Quatro novas espécies vegetais foram identificadas como hospedeiras *de A. bolboceridus e a* oeorrêneia da praga foi registrada em duas novas localidades. Este é o primeiro estudo da biologia de um repré sentante da tribo Agaocephalini no Brasil.

## Introduction

The white grubs are beetle larvae of the Melolonthidae (Insecta: Coleoptera) family *(sensu* Endrödi 1966; Morón 2001, 2004). This family comprises an important group of soil pests that cause severe damage to several types of crops by attacking their root system, decreasing productivity, and usually killing the plants (King 1984; Morón 1997b; Oliveira et al. 2007, 2008). The larvae usually develop in the soil, feeding on organic matter, including plant roots. They can also be found associated with some social insects (ants and termites) or developing in the cavities of live tree trunks and branches, beneath the bark or among the roots and in the auxiliary cavities of epiphytic plants (Morón 1997a, 2001). The Melolonthid adults can feed on all parts of angiosperms and gymnosperms, with a few species also preying on other insects. In Brazil, there are 1,008 species of the Melolonthidae family, belonging to 97 genera, whose larvae develop in the soil (Morón 2004). The genus *Aegopsis* Burmeister (Coleoptera: Melolonthidae: Dynastinae) belongs to the tribe Agaocephalini. It is exclusively found in the neotropics, and is currently known to include four species: *A. curvicornis, A. peruvianus, A. chaminadei*, and *A. bolboceridus* (Thomson).

*A. bolboceridus* was recently recorded as an agricultural pest that causes damage in areas of grain and vegetable production in the Brazilian Cerrado (Oliveira et al. 2008). During the rainy season, the larvae feed on the roots of a wide range of crops, often decreasing productivity and usually killing the plants (Oliveira et al. 2008; Oliveira 2009). This kind of damage has already been reported in at least 11 species of plants (Oliveira et al. 2008), and its presence has been confirmed in > 50% of the vegetable and corn production areas in central Brazil (Federal District and Goiás) (Oliveira 2009). The absence of reports on the occurrence of this pest until 2005 in central Brazil is attributable to the fact that only a few researchers study the soil pests in this region of the country.

The Brazilian Cerrado, where most of the food production in Brazil takes place (CONAB 2011), has a typically bimodal climate, with two well-defined seasons: a dry season (April to September), with an average annual rainfall of 185 mm, and a rainy season (October to March), with an average annual rainfall of 1,212 mm (Silva et al. 2008). This distinctive weather condition appears to have a decisive influence on the behavior and biology of insects within this biome (Gottsberger and Silberbauer-Gottsberger 2006; Oliveira and Frizzas 2008; Silva et al. 2011). In the rainy season, the temperature and water availability allow for widespread development of food resources for phytophagous insects (Silva et al. 2011). In the dry season, these resources are extremely scarce, which represents a serious obstacle to the development of many insect species. This type of environmental stress is a regular occurrence in the Cerrado, and these changes are normally predictable. The insects take advantage of this predictability by undergoing physiological and behavioral alterations that prepare them for the approaching season. Furthermore, it allows the hypothesis that insect species that have long cycles, as many Scarabaeoidea, synchronize their active stages of development with the rainy season in the Brazilian Cerrado and use specific strategies for survival (e.g. diapause) during the dry season.

A number of studies have been conducted to investigate the bioecology of Scarabaeoidea under field conditions (Jarvis 1966; Wiener and Capinera 1980; Lumbreras et al. 1990; Grunshaw 1992; Kuniata and Young 1992; Vitner 2000; Ansari et al. 2006). However, in Brazil, few studies have been conducted on this group of insects (Santos 1992; Silva and Loeck 1996), and no information exists on the biological aspects of the Agaocephalini (Pardo-Locarno and Morón 2006; Oliveira et al. 2008; Grossi 2009), which is essential for developing management strategies for *A. bolboceridus* in Brazil.

In addition to the biological aspects of *A. bolboceridus*, it is necessary to investigate the range of host plants and the geographical distribution of the pest. For example, the management strategies to be used can vary, depending on the host plants. Information about the geographical distribution of the pest can help monitor the expansion of *A. bolboceridus* to regions where the pest was not previously found. In addition, knowledge of behavioral characteristics, such as the swarming period and preferred oviposition sites, may allow for control measures, including capturing adults or modifying the characteristics of oviposition sites, making the area under cultivation less preferable to females.

The purpose of this work was to study the biological aspects (life cycle), behavioral aspects (oviposition behavior and swarming period), and host plants of *A. bolboceridus* in the field in the Brazilian Cerrado, and to relate these biological variables to the climatic characteristics of this biome as the basis for implementation of strategies to manage this pest.

## Materials and Methods

### Study area

Field investigations were conducted at the Taquara Rural Center (Chácara 70), Planalti-Planaltina/DF, Brazil (15° 37′ 12.86″ S; 47°3 1′ 49.10″ W; 1050 m) in an area of approximately 3 hectares. In this area, corn and at least 10 other species of vegetables are cultivated in spring/summer (October to March). In fall/winter, vegetables are cultivated using only sprinkler irrigation (April to September). Laboratory studies were conducted at Embrapa Cerrados (Planaltina/DF, Brazil) in climate chambers under controlled conditions (25 ± 2° C, 70 ± 23% RH, and 12:12 L:D photoperiod). Climatic variables, average monthly temperatures, total monthly rainfall, and average monthly relative humidity were recorded throughout the study period by the Embrapa Cerrados weather station, located approximately 20 km from the experimental area.

### Biological cycle

To study the occurrence and duration of biological stages of *A. bolboceridus*, samplings were conducted in the field every two weeks between November 2004 and November 2007 (72 sampling events in total). On each sampling occasion, 20 trenches (50 × 50 cm in area and 30 cm deep) were dug at random locations within the experimental area using a mattock (using trenches is a common method for locating the biological stages of Melolonthidae that develop in soil (Oliveira et al. 1996; Oliveira 1997)). The developmental stages of *A. bolboceridus* were recorded on site.

A subsample of the specimens found during each sampling event was transported to the laboratory, placed in plastic trays (40 cm long, 25 cm wide, and 8 cm high) containing moist, sterile soil from the experimental area, and maintained under controlled conditions. Trays containing bean plants (*Phaseolus vulgaris* L.), a natural host of *A. bolboceridus* (Oliveira et al. 2008), were used as food source for larval rearing (adults do not feed). For the egg, pre-pupae, and pupa phases, the trays contained only soil. The adult specimens obtained from these samples were used for specific taxonomic identification (see below).

### Determination of the number of instars

During sampling, a subsample of the *A. bolboceridus* larvae found was transported to the laboratory and boiled for approximately 2 minutes in an alcohol and water (1:1) solution (Almeida et al. 1998) to fixate the tissues. Next, the larvae were stored in bottles containing 70% alcohol. Later, the head capsule was measured using a micrometer attached to a stereomicroscope (Stemi SV6, Zeiss, www.zeiss.com). Measurements were performed on the dorsal region of the head of the larvae between the bases of the antennae. These measurements were placed in a frequency distribution to visualize the number of larval instars. To verify the pattern of growth during the larval stage, the mean measurement of the head capsule in each instar defined (response variable) was plotted against the number of larval instars (predictor variable), and a linear regression model, using the SAS statistical package (PROC REG; SAS Institute 2001), was established to verify the geometric pattern of growth. According to Dyar's rule (Dyar 1890), any deviation from this line would indicate that some instar was absent (Parra and Haddad 1989; Cunha et al. 1998). The growth rate was determined by dividing the mean measurement of the head capsule in the next instar by the mean measurement of the head capsule in the previous instar.

### Swarming period

Adults of *A. bolboceridus* are attracted to light. To evaluate the flight periods of adults, a light trap was installed at the center of the experimental area for weekly collections of adults between August 2006 and December 2007. Light traps provide reliable information on changes in the real size of populations of many species of night-flying insects (Wolda 1978) when their activity patterns are measured over time. The trap, similar to the INTRAL model, was made of metal and was formed by 4 fins arranged vertically into 2 perpendicular planes. A lamp was placed between the fins. This set was fixed to a metal plate (top) and a metal funnel (bottom). A F15T12 Black Light 350 fluorescent tube (Havells Sylvania Brazil Ltd, www.havellssylvania.com) that was powered by a 12V-60 Ah automotive battery (Cral Batteries Ltd., www.cral.com.br) and was coupled to a collection vessel containing alcohol and water (1:1) was used. The trap was fixed to a metal pole with a height of approximately 2 m, and the light remained on for 14 hr (18:00–08:00). The collected insects were transported to the laboratory, where *A. bolboceridus* specimens were separated from the other insects under a stereomicroscope.

The number of *A. bolboceridus* adults collected each month was correlated with the data of temperature, rainfall, and relative humidity, using the SAS statistical package (PROC CORR; SAS Institute 2001). The correlation was established for only the months in which the presence of adults in the light trap was detected.

### Preferred oviposition sites

Previous studies have shown that some members of the Melolonthidae select areas with plants taller than the surrounding vegetation as aggregation sites at which to mate and place eggs nearby (Garcia et al. 2003; Oliveira and Garcia 2003). In October and November 2005 and 2006, studies were undertaken to assess the oviposition sites of *A. bolboceridus* with respect to the presence of taller plants (> 0.80 m in height) such as *Brachiaria decumbens* Stapf cv Basilisk, *B. plantaginea* (Link) Hitch, *Panicum maximun* Jacq, and *Emilia sonchifolia* (L.) DC, and with respect to surrounding vegetation with presence of smaller plants (< 0.15 m in height) that had sparse distribution within the experimental area, such as *Tridax procumbens* L., *Bidens pilosa* L., *Sida santaremnensis* Monteiro, *Amaranthus hybridus* L., *Commelina benghalensis* L., and *Rhynchelytrum repens* (Willd.) CE Hubb. Samples were collected weekly from 40 random points within the experimental area; 20 points were located in areas with taller plants, and 20 points were located in areas with smaller plants. Trenches (50 × 50 cm in area and 30 cm deep) were excavated at each sampling point, and the number of eggs was recorded on site. A subsample of the eggs obtained was taken to the laboratory and maintained in Petri dishes (9 cm in diameter and 1.5 cm high) containing moist, sterilized soil, and covered with a perforated plastic film. After hatching, the larvae were reared until adult emergence to confirm their identification. The data on the number of eggs collected from the areas with taller plants and without vegetation were compared through *t*test analysis using the SAS statistical package (PROC TTEST; SAS Institute 2001).

### Host plants

In addition to the experimental area, other locations in Brazil that recorded attacks/damage by *A. bolboceridus* were visited, and the attacked plant species were identified. Farms showing symptoms of *A. bolboceridus* attacks were identified with the help of extension workers from the Technical Assistance and Rural Extension Company and a private crop consultant. The larvae found among plants with symptoms of attacks were transported to the laboratory and reared until adult emergence to confirm their identification.

### Taxonomic identification

For specific taxonomic identification, immature specimens collected during the study were reared in the laboratory until adult emergence and, together with the adults collected in the field, were compared with specimens deposited in the Embrapa Cerrados entomological collection that were previously identified by Dr. Miguel Angel Morón (Red de Biodiversidad y Sistemática, Instituto de Ecologia, A.C., Xalapa, Veracruz, Mexico) (see Oliveira et al. 2008). Identification was based on an examination of the external morphological characteristics and the genitalia. *Voucher* specimens of the studied material were deposited in the entomological collections of the Embrapa Cerrados, “Luiz de Queiroz” College of Agriculture (ESALQ) (Piracicaba/SP, Brazil) and Instituto de Ecologia, A.C. (IEXA) (Xalapa, Veracruz, Mexico).

## Results

### Biological cycle

**Figure 1.**
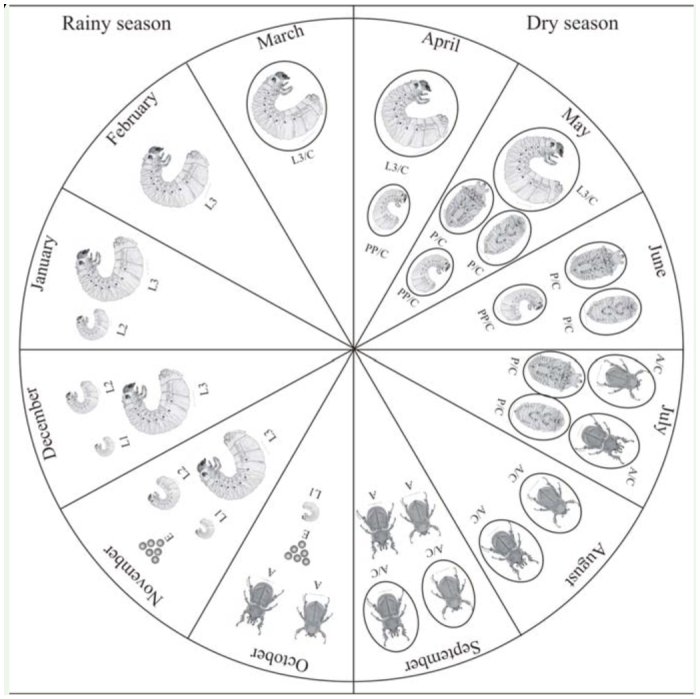
Biological cycle of *Aegopsis bolboceridus* and temporal distribution of its developmental stages based on soil samples collected between November 2004 and November 2007 at Planaltina/DF. A: adult; E: egg, L1 : first-instar larva; L2: secondinstar larva; L3: third-instar larva; L3/C: third-instar larva in diapause within the pupal chamber; PP/C: pre-pupa within the pupal chamber; P/C: pupa within the pupal chamber; A/C: adult within the pupal chamber. High quality figures are available online.

**Figure 2.**
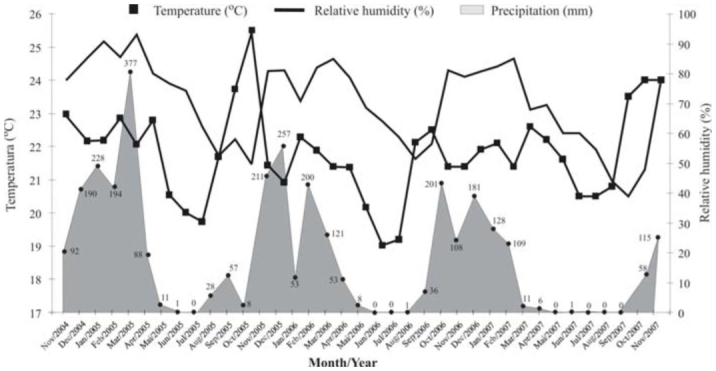
Average monthly temperature (°C), monthly precipitation (mm), and relative humidity for each month (%) from November 2004 to November 2007 at Planaltina/DF. High quality figures are available online.

The results of this study showed that *A. bolboceridus* undergoes a univoltine cycle, with approximately a 10-month period from egg to emergence of sexually immature adults, and approximately 12 months until the appearance of active adults (Figure 1). *A. bolboceridus* eggs were found in the field during October and November, soon after the onset of the rainy season (Figure 2), with 95.5% of the eggs found in October (Figure 1; Table 1). The eggs were isolated, but very close to each other, usually within a small chamber made of soil constructed by the female.

**Figure 3.**
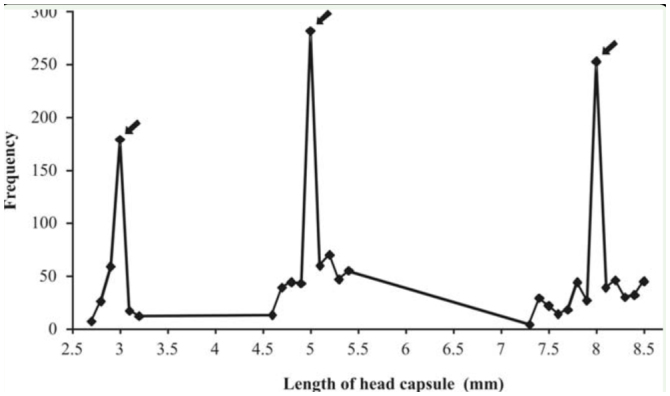
Frequency distribution of head capsules of *Aegopsis bolboceridus* larvae collected in the field in 2004, 2005, 2006, and 2007 at Planaltina/DF. The arrows indicate the three instars characterized by head capsule widths with higher frequencies. High quality figures are available online.

**Larval phase.** The larval phase was the longest among the developmental stages of *A. bolboceridus*, and larvae were found in different instars over a period of 8 months from October to May (Figure 1; Table 1). The head capsule measurements obtained for *A. bolboceridus* suggested the existence of three instars, with measurements of approximately 2.97 ± 0.016 mm (mean ± SEM) for the first instar, 5.04 ± 0.021 mm for the second instar, and 8.00 ± 0.035 mm for the third instar. The growth rate was 1.7 from the first to second instar and 1.6 from the second to third instar, following Dyar's rule (Dyar 1890). There was no overlap of 95% confidence intervals between the instars (Table 2). Linear regression analysis provided the following equation: y = 0.3067 + 2.515X (R^2^ = 0.989; n = 1,556). This indicates a geometric growth for the three larval instars, determined following Dyar's rule. In addition, the frequency distribution curve of the width of the head capsules indicates the presence of three instars (Figure 3).

First-instar larvae were found in the field between October and December, and 62.7% of these larvae were observed in November. Second-instar larvae were found from November to January, also with a peak in November (63.2%). Third-instar larvae were found for seven months, between November and May; however, from February on, only this instar was observed (Figure 1; Table 1). In March and April, third-instar larvae were commonly found beginning the construction of small elliptical-shaped clay chambers, known as pupal chambers, with internal dimensions ranging in length from 2.0 cm to approximately 4.0 cm.

**Table 1.**
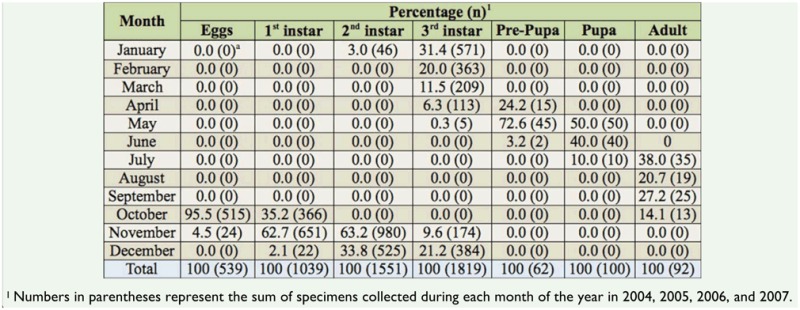
Relative percentage of developmental stage/instar occurrence of *Aegopsis bolboceridus* in relation to the month of the year and total number of specimens collected each month via soil samples (50 × 50 × 30 cm) in 2004–2007 at Planaltina/DF.

**Pre-pupa and pupa.** Specimens in the prepupal stage were observed between April and June, predominately in May (72.6%) (Table 1). At this stage, the specimens were much smaller than third-instar larvae, exhibiting a wrinkled body with an opaque white color, and dorso-ventral flattening in the terminal region of the body. The pre-pupae measured approximately 3.6 cm while the third-instar larvae reached 8.7 cm. Pupae were found in the field from May to July, with the greatest number of specimens being collected in May (50.0%) (Table 1). The pre-pupal stage appears to be relatively short (approximately one month), as the first pre-pupae were found in April and the first pupae in May. The pupal stage is assumed to be slightly longer because the first specimens in this phase were found in May, and adult emergence was observed starting in July (Figure 1; Table 1). These two stages, pre-pupa and pupa, occur within the pupal chambers (Figure 1).

**Adults.** During soil sampling, adults were found between July and October; they were observed (inactive) inside the pupal chambers until August and outside of the chambers (ac-tive) in September and October. Swarming *A. bolboceridus* adults were captured in the months of September (n = 3 for 2006, n = 2 for 2007) and October (n = 44 for 2006, n = 63 for 2007) using a light trap. The peak of the adult population always occurred in October, the same period when eggs were most commonly found in the soil (Table 1). This suggests that soon after the swarming period begins, mating occurs and egg-laying also starts.

It was observed that both sexes of *A. bolboceridus* can fly. Sexual dimorphism was evident, with the presence of one thoracic and two cephalic horns being observed in males. Feeding by adults was not observed. Adults were found above ground only at night, when some were observed mating, which suggests that males and females leave the soil for mating and dispersal. The period of SeptemberOctober, when active *A. bolboceridus* adults were collected, is the same timeframe as the onset of the rainy season in the brazilian Cerrado (Figure 2). A significant correlation was observed between the number of adults captured and increased rainfall (2006: R^2^ = 0.977, *p* = 0.0227; 2007: R^2^ = 0.984, *p* = 0.0158).

### Preferred oviposition sites

The *A. bolboceridus* females showed a clear oviposition preference for areas with the presence of taller plants. In 2005, an average of 13.76 ± 2.65 (mean ± SEM) (n = 1,376) eggs was registered at sampling points with taller plants, and 0.7 ± 0.24 (n = 66) eggs on smaller plants. In 2006, an average of 14.72 ± 2.70 (n = 1,472) and 0.5 ± 0.21 (n = 54) eggs was registered at sampling points with taller and smaller plants, respectively (Table 3). Statistically significant differences were found between the oviposition sites studied (2005: *t* = -4.93, df = 98, *p* < 0.001; 2006: *t* = -5.24, df = 98, p < 0.001), as the number of eggs in areas with taller plants was 19 and 29 times higher than that of areas with smaller plants in 2005 and 2006 respectively.

**Table 3.**
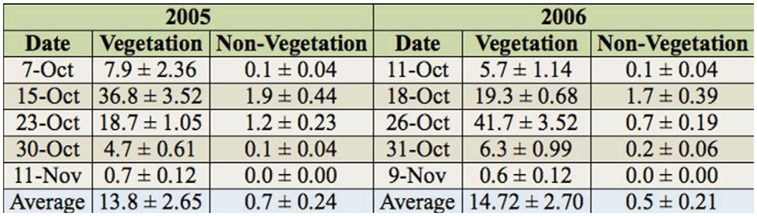
Average number (± SEM) of *Aegopsis bolboceridus* eggs per soil sample (50 × 50 × 30 cm) collected in October and November of 2005 and 2006 at Planaltina/DF from points with and without vegetation presence.

**Table 2.**
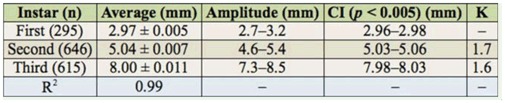
Average head capsule width (± SEM), head capsule amplitude variation, confidence interval (Cl), and growth rate (K) for *Aegopsis bolboceridus* larvae collected in the field in 2004–2007 at Planaltina/DF.

### Host plants

In this study, *A. bolboceridus* was found to be present in corn *(Zea mays* L.) at two new locations, Luziânia/GO and Planaltina/DF, as well as in four new host plants: strawberry *(Fragaria* × *ananassa* Duch), gilo *(Solanum gilo* Raddi), tomato (*Lycopersicon esculentum* Mill), and beet (*Beta vulgaris* L.). The attack on strawberry and gilo occurred at a new location, Brazlândia/DF (Table 4).

## Discussion

The results demonstrated that *A. bolboceridus* presented a relatively long biological cycle (about 1 year) with bioecological features highly adapted to the climatic conditions of the Cerrado of Central Brazil, especially with regard to the availability of water and food. These results support the hypothesis that, in the Cerrado, species with a long cycle synchronize their life cycle with environmental conditions, presenting polyphagia, intense feeding activity, and accumulating reserves during the rainy season, and with larval diapause and adult inactivity during the dry season.

**Table 4.**
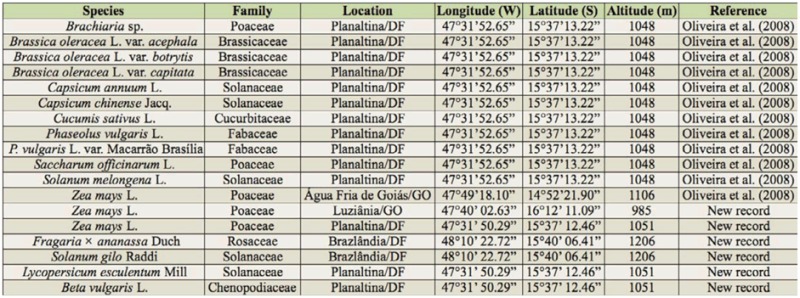
List of host species and their botanical families, collection sites, geographical coordinates, and reference to *Aegopsis bolboceridus* in central Brazil.

Similar to *A. bolboceridus*, most Melolonthidae, which are considered pests in Brazil, produce only one generation per year (Oliveira et al. 1996; Silva and Loeck 1996; Rodrigues et al. 2008a; Santos and Ávila 2009). Nesting behavior, similar to that of *A. bolboceridus*, has previously been observed for several other Melolonthidae subfamilies (King 1984; Kuniata and Young 1992; Santos 1992; Rodrigues et al. 2008a). Species that lay eggs in soil usually build oviposition chambers using a sticky secretion from the colleterial glands (Hayes 1929).

The presence of a larval phase that is longer than the other stages appears to be a common feature in most Scarabaeoidea (Hayes 1929; see Silva and Loeck 1996 for an extreme example). *A. bolboceridus* larvae were found moving freely in the soil and did not construct permanent galleries, as has been observed for other Dynastinae (Silva and Loeck 1996; Gassen 2000). The occurrence of three instars observed in *A. bolboceridus* is practically a rule for species of Scarabaeoidea (Hayes 1929; Ritcher 1957). However, some species of the genus *Pleocoma*, such as *P. minor* and *P. crinita*, may exhibit up to 13 instars (Ellertson and Ritcher 1959). Characteristics such as the construction of the pupal chambers (Ritcher 1957; King 1984) have also been observed in several species (Baucke 1965; Kuniata and Young 1992; Silva and Loeck 1996; Rodrigues et al. 2008a, b).

Studies have shown that the first rains occurring during the period from September to October at the beginning of the rainy season in the brazilian Cerrado act as a stimulus for the majority of insects to resume activity, especially those that live underground (Oliveira and Frizzas 2008; Silva et al. 2011). Therefore, the increase in soil moisture appears to provide the necessary stimulus for *A. bolboceridus* adults to leave the pupal chamber and initiate the swarming period, whose peak of the adult population occurs in October. Other Melolonthidae found in central Brazil, such as *L. fuscus* (Rodrigues et al. 2008a; Costa et al. 2009) and *L. suturalis* (Santos and Ávila 2009), also exhibit a peak adult population in October. Adults of *A. bol-boceridus* presented sexual dimorphism, as in other Dynastinae (Baucke 1965; Onore and Morón 2004; Céspedes and Ratcliffe 2010).

The preference for areas with taller plants by the female *A. bolboceridus* observed in this study suggests that these plants are used as aggregation sites, and that this strategy can be an attempt to increase the chance of survival of its larvae. This behavior may partially explain the characteristic attack pattern exhibited by larvae of this species in the field, which always occurs in patches. The concentration of eggs at certain sites in an area leads to a population of larvae that will consume the roots of their host plants within the site, usually in a radial design with irregular contours, forming such patches. In Brazil, *P. cuyabana* is an important soybean pest, and this species also shows a clear oviposition preference for areas with vegetation characterized by taller plants, which have been found to act as adult aggregation sites (Garcia et al. 2003).

*A. bolboceridus* has been reported to be a polyphagous pest. Oliveira et al. (2008) observed the occurrence of *A. bolboceridus* in at least eight genera of plants belonging to five plant families in the Federal District of Brazil and in the municipality of Agua Fria de Goiás/GO. Since 2005, when *A. bolboceridus* was first observed attacking vegetable crops in the Federal District (Oliveira et al. 2008), its importance as an agricultural pest in central Brazil (Federal District and Goiás) has grown, with reports of new hosts and occurrences at new locations (Oliveira et al. 2011). Here, *A. bolboceridus* was observed attacking new hosts and in new locations only in the Federal District and Goiás State, suggesting that its distribution is restricted to central Brazil.

In this study, it was observed that *A. bolboceridus* presents an active phase during the rainy season (between October and March) and an inactive phase (diapause syndrome), within the pupal chamber, during the dry season (from April to September) (Figure 1, 2). In central Brazil, due to the alternation between dry and rainy seasons (Silva et al. 2008), in areas where irrigation is not used, the cultivation of grain and other crops is restricted to the rainy season. Therefore, *A. bolboceridus* synchronizes the onset of its larval stage, which is the only stage at which the insect feeds, with summer plantings and ceases its activities during the early dry season, when food availability and soil moisture decrease. This synchrony has also been observed in another important soil pest, *P. cuyabana*, in areas of soybean production (Oliveira 1997). The inactive phase of *A. bolboceridus* appears to be governed by physiological and/or genetic factors, as adults enter the pupal chambers beginning in July and do not resume activity until September, even in areas under irrigation.

Effective strategies have not yet been developed to control *A. bolboceridus* in Brazil. The measures taken by agricultural producers to deal with pest-attack problems have included insecticide use and cultural practices. However, satisfactory results have not yet been obtained (Oliveira 2009). The biological and behavioral characteristics presented in this study may support the development of control measures for this pest by indicating biological stages and characteristics that will allow behavioral interventions to be employed with greater chance of success.

The results of our study suggest that the key period for adoption of control measures for *A. bolboceridus* is October-November, when the rains begin in the Cerrado (Figure 2) and most likely stimulate the onset of adult activity. Control measures, such as the use of insecticides, should be used during this period, when most *A. bolboceridus* larvae are in the firstinstar stage and are most likely to be more sensitive to insecticides. Cultural methods such as mass collection of adults by using light traps to reduce the pest population should be performed soon after the first rains in October, when most adults leave the soil for mating and dispersal. Weed control in the crop areas may also provide an alternative method for controlling the insect. The elimination of taller plants (weeds) before the beginning of the swarming period can forced. *bolboceridus* adults into searching for oviposition sites in other areas. In addition, the soil can be plowed in March and April to destroy the immature stages of *A. bolboceridus*. During this period, there is still moisture in the soil, allowing access to tractors and agricultural implements, coupled with the fact that the larvae are in diapause and, when exposed, cannot return to the soil and can die of dehydration or by predation. Additional studies, such as on natural enemies of *A. bolboceridus*, are still required to better understand the bioecology of this species. This is the first study about the biology of a representative of the tribe Agaocephalini in Brazil.

## References

[bibr01] Almeida LM, Ribeiro-Costa BCS, Marinoni L (1998). *Manual de cole ta, conservação, montagem e identificação de insetos*,.

[bibr02] Ansari MA, Casteels H, Tirry L, Moens M (2006). Biology of *Hoplia philanthus* (Coleoptera: Scarabaeidae), a new and severe pest in Belgian turf.. *Environmental Entomology*.

[bibr03] Baucke O (1965). Notas taxinômicas e biológicas sobre *Diloboderus abderus* (Sturm, 1826) Coleoptera-Scarabaeidae-Dynastinae.. *Revista da Faculdade de Agronomia e Veterinaria*.

[bibr04] Céspedes AA, Ratcliffe BC (2010). *Golofa clavigera* (Linnaeus, 1771) in Bolivia: a new country record (Coleoptera: Scarabaeidae: Dynastinae).. *Ecología en Bolivia*.

[bibr05] CONAB. Companhia Nacional de Abastecimento. (2011). *Acompanhamento da safra brasileira: grãos*. segundo levantamento, Novembre 2011. Companhia Nacional de Abastecimento..

[bibr06] Costa RB, Fernandes PM, Oliveira FS, Rocha MR, Morón MA, Oliveira LJ (2009). Captura de adultos de *Liogenys fuscus* (Coleoptera: Melolonthidae) com armadilha luminosa em área sob sistema de plantio direto.. *Bioscience Journal*.

[bibr07] Cunha MCI, Coscarón S, Bassi RMA (1998). Determinación de los estadios larvales de *Simulium* (Diptera, Simuliidae) de Paraná, Brasil.. *Acta Biológica Paranaense*.

[bibr08] Dyar H (1890). The number of moults in lepidopterous larvae.. *Psyche*.

[bibr09] Ellertson FE, Ritcher PO (1959). Biology of rain beetles, *Pleocoma* spp., associated with fruit trees in Wasco and Hood River Counties.. *Oregon State University Agricultural Experiment Station Technical Bulletin*.

[bibr10] Endrödi S (1966). Monographie der Dynastinae (Coleoptera: Lamellicornia) I. Teil.. *Entomologische Abhandlungen Museum Tierkunde*.

[bibr11] Garcia MA, Oliveira LJ, Oliveira MCN (2003). Aggregation behavior of *Phyllophaga cuyabana* (Moser) (Coleoptera: Melolonthidae): Relationships between sites chosen for mating and offspring distribution.. *Neotropical Entomology*.

[bibr12] Gassen DN (2000). Embrapa Trigo Comunicado Técnico Online 47.. *Os benefícios de corós em lavouras sob plantio direto*..

[bibr13] Gottsberger G, Silberbauer-Gottsberger I (2006). *Life in the Cerrado: a South American tropical seasonal ecosystem*,.

[bibr14] Grossi PC, Abadie EI, Grossi PC, Wagner PS (2009). Introduction.. *A field guide of the Dynastidae family of the south of South America*..

[bibr15] Grunshaw JP (1992). Field studies on the biology and economic importance of *Pachnoda interrupta* (Coleoptera: Scarabaeidae) in Mali, West Africa.. *Bulletin of the Entomological Research*.

[bibr16] Hayes WP (1929). Morphology, taxonomy, and biology of larval Scarabaeoidea.. *Illinois Biological Monographs*.

[bibr17] Jarvis JL (1966). Studies of *Phyllophaga anxia* (Coleoptera: Scarabaeidae) in the sandhills area of Nebraska.. *Journal of the Kansas Entomological Society*.

[bibr18] King ABS (1984). Biology and identification of white grubs (*Phyllophaga*) of economic importance in Central America.. *Tropical Pest Management*.

[bibr19] Kuniata LS, Young GR (1992). The biology of *Lepidiota reuleauxi* Brenske (Coleoptera: Scarabaeidae), a pest of sugarcane in Papua New Guinea.. *Journal of the Australian Entomological Society*.

[bibr20] Lumbreras CJ, Galante E, Mena J (1990). An ecological study of the dung beetle *Bubas bubalus* (Oliver, 1811)(Col. Scarabaeidae).. *Acta Zoologica Mexicana (nueva serie)*.

[bibr21] Morón MA (2001). Larvas de escarabajos del suelo em México (Coleoptera: Melolonthidae).. *Acta Zoologica Mexicana* (*nueva serie)*.

[bibr22] Morón MA, Salvadori JR, Ávila CJ, Silva MT (2004). Melolontideos edaficolas.. *Pragas de Solo no Brasil*..

[bibr23] Morón MA (1997a). Inventarios faunísticos de los Coleoptera Melolonthidae Neotropicais con potencial como bioindicadores.. *Giornale Italiano di Entomologia*.

[bibr24] Morón MA (1997b). White grubs (Coleoptera: Melolonthidae: *Phyllophaga* Harris) in Mexico and Central America. A brief review.. *Trends in Entomology*.

[bibr25] Oliveira CM (2009). Situação atual de corós rizófagos no Distrito Federal e municípios do entorno.. *Embrapa Cerrados Série Documentas* 248..

[bibr26] Oliveira CM, Frizzas MR (2008). Insetos de Cerrado: distribuição estacional e abundância.. *Embrapa Cerrados Boletim de Pesquisa e Desenvolvimento* 216..

[bibr27] Oliveira CM, Morón MA, Frizzas MR (2007). First record of *Phyllophaga* sp. aff. *capillata* (Coleoptera: Melolonthidae) as a soybean pest in the brazilian “Cerrado”.. *The Florida Entomologist*.

[bibr28] Oliveira CM, Morón MA, Frizzas MR (2008). *Aegopsis bolboceridus* (Coleoptera: Melolonthidae): an important pest on vegetables and corn in Central Brazil.. *The Florida Entomologist*.

[bibr29] Oliveira CM, Lopes RB, Moino A, Frizzas MR, Mertz NR, Almeida JEM (2011). Ocorrência natural de nematóides entomopatogênicos em larvas de *Aegopsis bolboceridus* (Thomson) (Coleoptera: Melolonthidae) no Brasil Central.. *Proceedings of the 12th Simpósio de Controle Biológico*..

[bibr30] Oliveira LJ (1997). *Ecologia comportamental e de interações com plantas hospedeiras em Phyllophaga cuyabana (Moser) (Coleoptera: Melolonthidae, Melolonthinae) e implicaçoes para o seu manejo em cultura de soja*..

[bibr31] Oliveira LJ, Garcia MA (2003). Flight, feeding and reproductive behavior of *Phyllophaga cuyabana* (Moser) (Coleoptera: Melolonthidae) adults.. *Pesquisa Agropecuária Brasileira*.

[bibr32] Oliveira LJ, Santos B, Parra JRP, Amaral MLB, Magri DC (1996). Ciclo biológico de *Phyllophaga cuyabana* (Moser) (Scarabaeidae: Melolonthinae).. *Anais da Sociedade Entomológica Brasil*.

[bibr33] Onore G, Morón MA (2004). *Dynastes neptunus* Quenzel (Coleoptera: Scarabaeidae: Dynastinae); description of the third instar larva and pupa, with notes on biology.. *The Coleopterist Bulletin*.

[bibr34] Pardo-Locarno LC, Morón MA (2006). Description of the third-instar larva and pupa of *Lycomedes hirtipes* Arrow (Coleoptera: Scarabaeidae: Dynastinae:Agaocephalini) with notes on its biology and distribution in Colombia.. *Proceedings of the Entomological Society of Washington*.

[bibr35] Parra JRP, Haddad ML (1989). *Determinação do número de ínstares de insetos*,.

[bibr36] Ritcher PO (1957). Biology of Scarabaeidae.. *Annual Review of Entomology*.

[bibr37] Rodrigues SR, Barbosa CL, Puker A, Abot AR, Ide S (2008a). Occurrence, biology and behavior of *Liogenys fuscus* Blanchard (Insecta, Coleoptera, Scarabaeidae) in Aquidauana, Mato Grosso do Sul, Brazil.. *Revista Brasileira de Entomologia*.

[bibr38] Rodrigues SR, Puker A, Abot AR, Barbosa CL, Ide S, Coutinho GV (2008b). Ocorrência e aspectos biológicos de *Anomala testaceipennis* Blanchard (Coleoptera, Scarabaeidae).. *Revista Brasileira de Entomologia*.

[bibr39] Salvadori JR, Silva MTB, Salvadori JR, Ávila CJ, Silva MT (2004). Coró-dotrigo.. *Pragas de Solo no Brasil*..

[bibr40] Santos B (1992). *Bioecologia de Phyllophaga cuyabana (Moser 1918) (Coleoptera: Scarabaeidae), praga do sistema radicular da soja [Glycine max (L.) Merrill, 1917]*..

[bibr41] Santos V, Ávila CJ (2009). Aspectos biologicos e comportamentais de *Liogenys suturalis* Blanchard (Coleoptera: Melolonthidae) no Mato Grosso do Sul.. *Neotropical Entomology*.

[bibr42] SAS Institute. (2001). *PROC user's manual*,.

[bibr43] Silva FAM, Assad ED, Evangelista BA, Sano SM, Almeida SP, Ribeiro JF (2008). Caracterização climática do bioma Cerrado.. *Cerrado: ecologia e flora*..

[bibr44] Silva MTB, Loeck AE (1996). Ciclo evolutivo e comportamento de *Diloboderus abderus* Sturm (Coleoptera: Melolonthidae) em condições de plantio direto.. *Anais da Sociedade Entomologica do Brasil*.

[bibr45] Silva NAP, Frizzas MR, Oliveira CM (2011). Seasonality in insect abundance in the “Cerrado” of Goiás State, Brazil.. *Revista Brasileira de Entomologia*.

[bibr46] Vitner J (2000). Field observations on the biology of *Copris lunaris* (Coleoptera: Scarabaeidae).. *Acta Societatis Zoologicae Bohemicae*.

[bibr47] Wiener LF, Capinera JL (1980). Preliminary study of the biology of the white grub *Phyllophaga flmbripe s* (LeConte) (Coleoptera: Scarabaeidae).. *Journal of the Kansas Entomological Society*.

[bibr48] Wolda H (1978). Seasonal fluctuations in rainfall, food and abundance of tropical insects.. *Journal of Animal Ecology*.

